# Comparison of Cheese Aroma Intensity Measured Using an Electronic Nose (E-Nose) Non-Destructively with the Aroma Intensity Scores of a Sensory Evaluation: A Pilot Study

**DOI:** 10.3390/s21248368

**Published:** 2021-12-15

**Authors:** Kouki Fujioka

**Affiliations:** Research Center for Agricultural Information Technology, National Agriculture and Food Research Organization, 1-31-1 Kannondai, Tsukuba, Ibaraki 305-0856, Japan; fujioka_koki@affrc.go.jp

**Keywords:** cheese, electronic nose (e-nose), sensory evaluation, non-destruction, aroma

## Abstract

Cheese aroma is known to affect consumer preference. One of the methods to measure cheese aroma is the use of an electronic nose (e-nose), which has been used to classify cheese types, production areas, and cheese ages. However, few studies have directly compared the aroma intensity scores derived from sensory evaluations with the values of metal oxide semiconductor sensors that can easily measure the aroma intensity. This pilot study aimed to investigate the relationship between sensory evaluation scores and e-nose values with respect to cheese aroma. Five types of processed cheese (two types of normal processed cheese, one type containing aged cheese, and two types containing blue cheese), and one type of natural cheese were used as samples. The sensor values obtained using the electronic nose, which measured sample aroma non-destructively, and five sensory evaluation scores related to aroma (aroma intensity before intake, during mastication, and after swallowing; taste intensity during mastication; and remaining flavor after swallowing (lasting flavor)) determined by six panelists, were compared. The e-nose values of many of the tested cheese types were significantly different, whereas the sensory scores of the one or two types of processed cheese containing blue cheese and those of the natural cheese were significantly different. Significant correlations were observed between the means of e-nose values and the medians of aroma intensity scores derived from the sensory evaluation testing before intake, during mastication, and after swallowing. In particular, the aroma intensity score during mastication was found to have a linear relationship with the e-nose values (Pearson’s R = 0.983). In conclusion, the e-nose values correlated with the sensory scores with respect to cheese aroma intensity and could be helpful in predicting them.

## 1. Introduction

Cheese is a dairy product that is widely consumed, particularly in Europe and North America. In 2018, approximately 2 and 1.7 million tons of cheese were consumed in Germany and France, respectively [1]. Approximately 353,000 tons of cheese were consumed in Japan (FY2018), of which approximately 210,000 tons were natural cheese and 143,000 tons were processed cheese [2]. Many people in Japan prefer processed cheese [3]. Arai et al. [4] investigated the preference for six types of cheese in Japan (including both natural and processed cheeses) in 32 women aged 19–20 years and 25 women aged 20–60 years; 86.7% and 24.0% of the women chose processed cheese as their first or second preferences, respectively.

Cheese has a unique aroma which involves more than 600 types of volatile components [5] that are known to affect preference and sensory evaluation. For example, the surface color and flavor—including taste and aroma—affect the preference for cheddar cheese [6], and preference scores and acetic acid flavor intensity in sensory evaluation can be predicted based on aroma components and viscoelasticity [7]. Moreover, a study in France reported that 60% of people who do not like cheese are repelled by its aroma, visual aspects, and texture [8]. Thus, cheese aroma is an important factor in determining the preference and flavor characteristics of cheese.

One way to analyze the aroma is by using an electronic nose (e-nose) [9,10,11,12]. E-nose is often composed of a sensor/sensor array, such as metal oxide semiconductors and polymers that respond to volatile components [12]. In addition, in some cases, the e-nose includes a method of analyzing components by gas chromatography (GC) or gas chromatography-mass spectrometry (GC-MS) and classifying the samples from the components’ pattern. Using e-noses, the approximate concentration of volatile components or aroma intensity can be estimated from the change in the electric resistance due to the contact of the volatile components [9]. E-noses have been applied to measure volatile components in several foods [9,13,14], for example, in comparing apple juices [15], bread [16], potatoes [17], and herb and fruit essential oils [18]; and describing coffee aromas similar to how wine experts describe wine [19].

E-nose systems have also been used to classify cheese type, production area, and ripening period [20]. Flamengo, Brie, Gruyère, and Mozzarella cheese have been classified [21]. In the same study, Brie cheese was classified according to their origin. E-nose systems have also been used to distinguish the regions where cheeses are produced, for example, Swiss Emmental cheese was compared with Emmental cheese from other regions [22]. Oscypek cheese marked with the European Union’s protected designation of origin status and oscypek-like cheese have also been classified [23,24]. Finally, e-noses have been used to determine the aging period for Pecorino [25], Parenica [26], Emmental [27], Cheddar [28], Crescenza [29], Danish blue [30,31], and Sir iz mišine cheeses [32]. Thus, methods to measure cheese aroma using e-noses for classifying cheese type, production area, and ripening period have been investigated.

There is an opinion that consumers need a way to quickly interpret and classify flavor, shelf life, and production method, being a purpose for which e-noses can be applied [10]. At present, since the e-nose technique for quick multitasking is still under development, sensory evaluation can be helpful for their needs. However, since sensory evaluation is affected by factors such as physical condition and olfactory fatigue, there are sometimes problems with reproducibility and the number of samples that can be measured in one day. In addition, 75–100 h of training is required to develop a panel for evaluating the flavor of cheese [33]. Therefore, if the sensory evaluation scores can be easily predicted by the values obtained using metal oxide semiconductor sensors, then the burden on the sensory evaluation panel can be reduced by narrowing the number of measurement samples. This makes it possible to meet the needs of consumers with a simple method of flavor evaluation.

**Figure 1:**
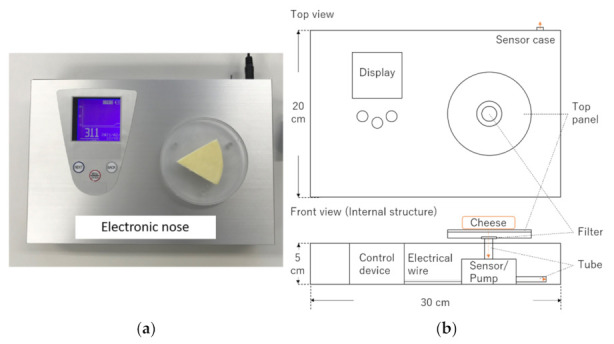
Electronic nose for sample bottom aroma: (**a**) measurement of a cheese sample; (**b**) diagram of the electronic nose.

In this study, to clarify the relationship between the e-nose sensor values and the aroma intensity and related scores in sensory evaluation, the values and scores for cheese aroma were compared. For this purpose, six cheeses (processed and natural cheeses) were measured using a non-destructive e-nose that measures sample bottom aroma (Figure 1), and evaluated by six panelists. This study was a pilot study to investigate the relationship between sensor values and aroma intensity-related scores in sensory evaluation during cheese consumption.

## 2. Materials and Methods

### 2.1. Cheese Samples

Five kinds of processed cheeses (A–C, E, and F) and one kind of natural cheese (D) were chosen as cheese samples for e-nose measurement and sensory evaluation of aroma-related scores (Table 1). These cheeses are commercially available in Japan. Among the processed cheeses, two were normal type processed cheese (A, B), one contained aged cheese (C; aged cheese type), and two contained blue cheese (E, F; blue cheese type). The type of natural cheese (D) used was relatively weak in flavor. The cheeses were stored in a refrigerator until measurement or evaluation.

### 2.2. Electronic Nose Measurements

For the electronic nose, a commercially available handy odor sensor (POLFA, Karumoa Co., Ltd., Tokyo, Japan) was modified, and a new device capable of non-destructive measurement of the sample aroma from bottom (Figure 1) was used. The e-nose device was manufactured under commission by ARAKI SANGYO CO., LTD., (Osaka, Japan). Permission was obtained from Karumoa Co., Ltd. to modify the POLFA odor sensor as a part of the mechanism of the e-nose device.

The commercially available cheeses were left at room temperature (22–23 °C) before aroma measurements (30 min), and then placed on the top panel of the sensor device (Figure 1). The cheese aroma from the sample bottom was collected through the hole in the top panel and was measured continuously with a sensor (metal oxide semiconductor) until the value was stabilized. After the measurement, the cheese sample was removed from the top panel and placed at room temperature. After cleaning the sensor with room air, the next sample was placed on the top panel and measured. The cheese sample aroma was measured again after kept for another 60 min (total 90 min) under room temperature. Three different samples were prepared per cheese type. The flow rate of the POLFA odor sensor was around 350 mL/min according to the manufacturer’s manual, and the rate of the electronic nose for the sample bottom aroma was estimated to be 300–400 mL/min. The sensor values when the value stabilized (near peak values) were used as the aroma intensity. The average intensity of the blank (room air) measured on the same day was 102.7 (*n* = 3). The dates of e-nose measurement and sensory evaluation were the same, but the locations were different.

### 2.3. Sensory Evaluation

**Figure 2:**
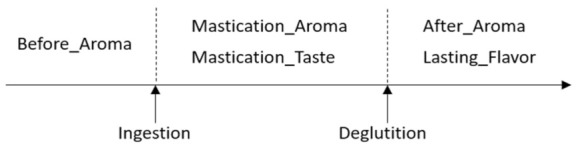
Diagram of sensory evaluation for cheese aroma intensity scores and related scores.

The sensory evaluation of cheese was performed by six women, 20–59 years old (panelists who passed a taste and smell test), who agreed to participate and provided informed consent. The evaluation test was performed once as a pilot study. Six cheese samples were placed at room temperature (23 °C) 20 to 30 min before the evaluation and were cut into small pieces. These six cheese samples were served one by one in the same order for all panelists. The evaluation was then carried out at room temperature without revealing the product name. The panelists could drink water to clean their mouth (not compulsory), and they could return to take previous cheese samples in the evaluation. Five characteristic evaluations were set: the aroma intensity before intake (Before_Aroma), during mastication (Mastication_Aroma), and after swallowing (After_Aroma), the taste intensity of the flavor during mastication (Mastication_Taste), and the persistence of flavor after swallowing (Lasting_Flavor), which were parameters expected to be related to aroma intensity (Figure 2). The characteristic evaluation was scored in seven grades from −3 (very weak) to +3 (very strong) in aroma and taste intensities or −3 (very short) to +3 (very long) in lasting flavor. To suppress the variations in the scores, evaluation was performed based on processed cheese A (all the scores of cheese A = 0).

The sensory evaluation method used in this study was reviewed and approved by the Ethics Committee of the National Agriculture and Food Research Organization (No. R02-Research Center for Agricultural Information Technology 01, 3 December 2020). The sensory evaluation test was performed at House Food Analytical Laboratory Inc., Chiba, Japan.

### 2.4. Statistical Analysis

Statistical analyses were performed using SPSS version 28 (IBM Corporation, New York, NY, USA) and Microsoft Excel (Microsoft, Seattle, WA, USA). The data of the e-nose device were confirmed to be normal using the Shapiro–Wilk normality test. After the one-way ANOVA, the e-nose data of each sample were compared using Tukey’s HSD test. For the comparison of placement time at room temperature, the e-nose values of 30 and 90 min were compared using paired *t*-test. In the sensory evaluation data, as the data of some samples did not show normality, each sample was compared using the Bonferroni/Dunn test after the Kruskal–Wallis test. Central tendencies, the mean of e-nose values and the median of sensory evaluation scores, in the six cheeses, were confirmed to be normal. Correlation tests were performed to determine the correlation coefficients (Pearson’s correlation coefficient and Spearman’s rank correlation coefficients) between the e-nose values (mean) and the sensory evaluation scores (median). Statistical significance was set at 0.05.

## 3. Results

### 3.1. Sensory Evaluation of Cheeses

**Figure 3:**
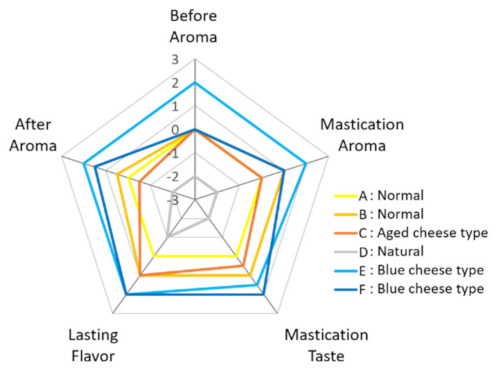
Aroma intensity scores and related sensory scores of the 6 cheese samples in sensory scores (Median).

**Figure 4:**
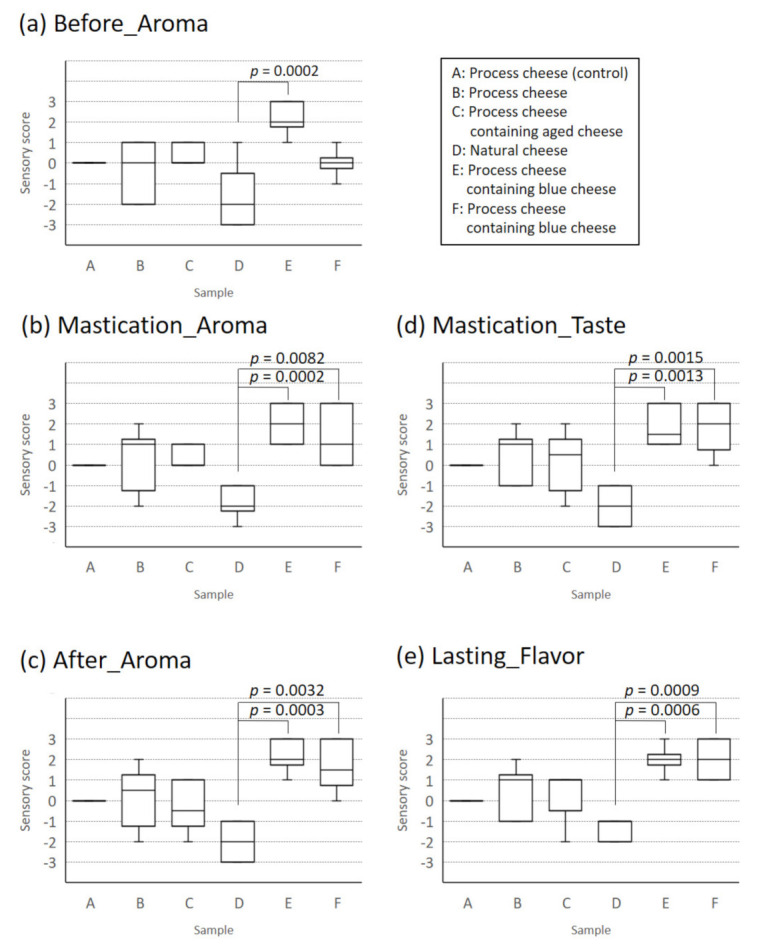
Comparison of aroma intensity scores and related sensory scores for 6 cheeses: (**a**) Before_Aroma; (**b**) Mastication_Aroma; (**c**) After_Aroma; (**d**) Mastication_Taste; (**e**) Lasting_Flavor.

The aromas of the six types of commercially available cheese were evaluated by sensory evaluation (by an analytical panel, six panelists). In the sensory evaluation, five characteristics were evaluated: the aroma intensities before intake, during mastication, after swallowing; the taste intensity during mastication; and the lasting flavor (Figure 2). First, to understand the characteristics of cheeses, the median score of the evaluation items was plotted on a radar chart and boxplot (Figure 3 and Figure 4).

Natural cheese (D) tended to have lower scores than other cheeses in all characteristic evaluations, whereas blue cheese types (E) and (F) tended to have higher scores. However, for blue cheese type (F), the aroma intensity before intake was the same as that of the other three types of processed cheese (A–C). The aroma intensity of cheese (F) during mastication was the same as that of the normal type (B). As for other scores, the scores of normal types (A), (B), and aged cheese type (C) were plotted between natural cheese (D) and blue cheese types (E) and (F). The significance test revealed a significant difference between (D) and (E) in the aroma intensity before intake (Figure 4). In addition, significant differences were observed between (D) and (E) and between (D) and (F) in the aroma intensity during mastication, aroma intensity after swallowing, taste intensity during mastication, and lasting flavor (Figure 4).

When the correlations in sensory evaluation scores were investigated, significant correlations were found in the intensity of aroma before intake, during mastication, and after swallowing (Table 2). In contrast, no significant correlation was found between the aroma before intake and the taste intensity during mastication, and between the aroma before intake and that persisting after swallowing. In addition, the highest correlation was found between the aroma intensity score during mastication and the aroma intensity score after swallowing, and the taste intensity during mastication and lasting flavor after swallowing.

### 3.2. E-Nose Values of Cheeses

**Figure 5:**
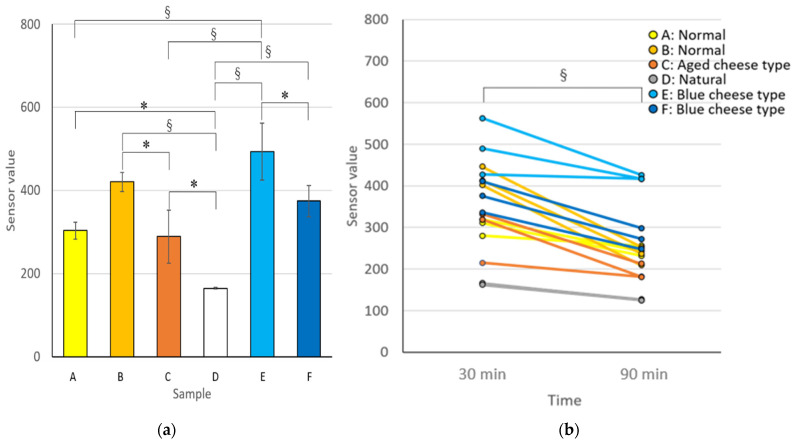
Electronic nose sensor values for 6 cheeses (*n* = 3). The Tukey HSD test (**a**), and paired *t*-test (**b**), were used for comparison (*: *p* < 0.05; §: *p* < 0.005). Error bar: standard deviation. (**a**) Sensor values of cheese samples (30 min); (**b**) comparison of placement time between 30 and 90 min at room temperature.

As for the e-nose value, the mean values were calculated by measuring three samples in each cheese after placing the cheese at room temperature for 30 min, similar to the sensory evaluation (Figure 5a). Blue cheese type (E) had the highest value, followed by normal type (B), blue cheese type (F), normal type (A), aged cheese type (C), and natural cheese (D). Cheese (D) was significantly different from all other cheeses (Figure 5a). In the sensory evaluation, blue cheese type (F) had the same score as normal cheese (B) in the aroma intensity before intake and during mastication, and a similar result was obtained in the e-nose sensor values. To investigate the effect of placing time at room temperature on the e-nose value, the e-nose values for 30 min were compared with those left at room temperature for 90 min (Figure 5b). The sensor values of cheeses placed at room temperature for 90 min were significantly lower than those of cheese placed at room temperature for 30 min (*p* = 0.000003). The mean differences between 30 and 90 min were the smallest in D (38.7) and the largest in B (187.3).

### 3.3. Comparison of Sensory Evaluation Scores and E-Nose Values

**Figure 6:**
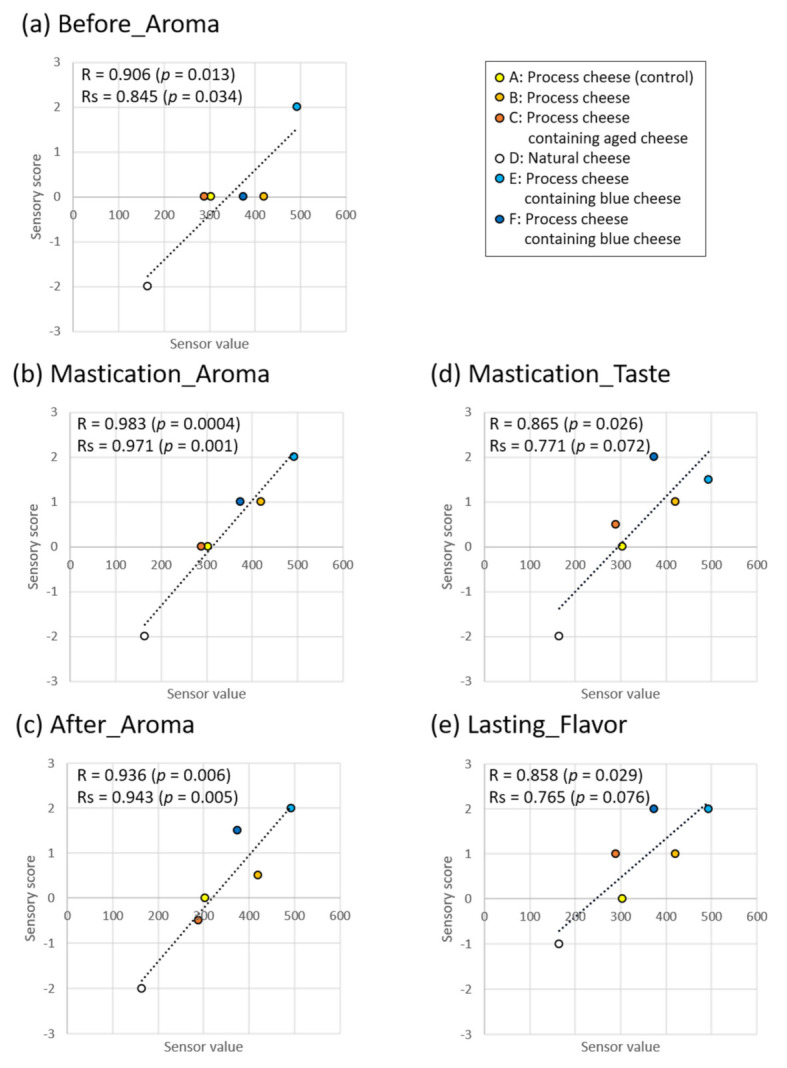
Correlation of electronic nose sensor values (mean values in Figure 5a) and sensory scores (median values in Figure 4). R: Pearson’s correlation coefficient. Rs: Spearman’s rank correlation coefficient. (**a**) Before_Aroma; (**b**) Mastication_Aroma; (**c**) After_Aroma; (**d**) Mastication_Taste; (**e**) Lasting_Flavor.

When investigating the correlation between the mean e-nose values (placed for 30 min, Figure 5a) and the median of sensory evaluation scores (Figure 4), the correlation coefficients were 0.858–0.983 in Pearson’s correlation coefficients (R) for linear correlation, and 0.765–0.971 in Spearman’s rank correlation coefficients (Rs) (Figure 6). For predicting aroma intensity scores in sensory evaluation using the e-nose, both correlations with sensory scores are important.

As for the aroma intensity before intake, during mastication, and after swallowing, blue cheese type (E) had the highest e-nose sensor value and sensory evaluation score (e-nose: 493.3, sensory score: 1.5–2.0), and natural cheese (D) had the lowest sensor value and evaluation score (e-nose: 164.3, sensory score: −1.0 to −2.0). The sensor values of the other cheeses ranged from 288.7 to 420.3, but their aroma intensity scores before intake were zero (Figure 6a). In contrast, a nearly linear relationship was observed between the sensor values and aroma intensities during mastication (R = 0.983, Rs = 0.971; Figure 6b). For the aroma intensities after swallowing (Figure 6c), normal type (B) and blue cheese type (F) deviated from the linear relationship slightly; however, a strong correlation was still observed (R = 0.936, Rs = 0.943).

In addition, in the Spearman’s rank correlation coefficients, no significant correlation was found between the e-nose value and the taste intensity during mastication (Figure 6d), nor between the e-nose values and the scores of lasting flavor after swallowing (Figure 6e), although significant correlation was found in Pearson’s correlation coefficients.

## 4. Discussion

Previous studies have performed sensory evaluation of cheese volatile components. For example, models to estimate preference scores from volatile components of cheddar cheese [3], and sensory evaluation values from volatile components and viscoelasticity [7] have been reported. Sensory evaluation and cheddar cheese classification by sensors using metal oxide semiconductor sensors have also been compared [34,35]. However, few studies have directly compared the cheese aroma intensity scores of sensory evaluation with the sensor values obtained using semiconductor sensors. In this study, the sensor values were significantly correlated with the aroma intensity scores of the sensory evaluation (Figure 6); the sensor values and aroma intensity score during mastication were almost linear (Figure 6b). These results suggest that the e-nose can be used to predict the aroma intensity of cheese during mastication.

Significant differences were observed in the e-nose values among several cheeses (Figure 5a), but the aroma intensity scores before intake in the sensory evaluation changed only for blue cheese type (E) and natural cheese (D) (Figure 3 and Figure 4a). It may be necessary to exceed the concentration threshold to recognize the different level in cheese aroma by a human. That is, even if there is a difference in the concentration of volatile components, the score is constant until the difference exceeds the threshold value; when the difference exceeds the threshold value, the difference in aroma intensity is strongly recognized. In addition, since the concentration of volatile components is relatively low before intake, it is difficult to exceed the threshold for recognizing the difference in scores; hence, the four types of cheese have the same score. On the contrary, during mastication, a considerable amount of volatile components contained in cheese are released and, therefore, the components exceed the threshold in several cheeses, allowing the aroma difference of cheeses to be recognized easily. With respect to other characteristics, in the Spearman’s rank correlation coefficients, no significant correlation was found between the e-nose values and the scores of taste during mastication or lasting flavor after swallowing in the sensory evaluation, although significant correlation was found in Pearson’s correlation coefficients (Figure 6d,e). Therefore, to predict taste intensity with high accuracy, it may be better to supplement data measured by other e-nose sensors or other measurement methods, such as taste sensors.

Regarding the correlation of sensory evaluations, the aroma intensity during mastication and the aroma intensity after swallowing were found to be significantly correlated with all other sensory evaluation values (Table 2). The aroma intensity during mastication and after swallowing are important to evaluate cheese flavor. In addition, the aroma intensity during mastication and after swallowing are highly correlated. Therefore, predicting the characteristics of aroma during mastication could be useful in evaluating the flavor of cheese. In contrast, lasting flavor was more highly correlated with taste intensity during mastication than with aroma intensities (Table 2). Thus, taste intensity is also important for evaluating the flavor of cheese.

This study shows that placement time at room temperature after removing the cheese from the refrigerator affects the aroma intensity. In previous studies, using the same type of cheeses, e-noses have been applied to compare maturity over several months [25,26,27,28,29,30,31,32]. However, few studies have investigated the aroma changes in the cheeses over a short period after removing them from the refrigerator. In this study, using a non-destructive measurable e-nose, it was possible to measure and compare the aroma intensity of the same cheese in a shorter time (30 and 90 min). There was a difference in the e-nose values (Figure 5b, mean difference: 38.7–187.3). In a study of cheddar cheese [36], tested serving temperatures (5, 12, and 21 °C) had no effect on flavors other than acidity. Therefore, the cheese volatile components in this study may be reduced by time-dependent releasing. Although the placement times of 30 and 90 min were not compared in the sensory evaluation, when there was a difference of approximately 100 to 200 in the aroma sensor value, it was assumed that the score during mastication would change by approximately 1–2 (Figure 6). For consumers who prefer a stronger flavor, it may be better to eat cheese that has been left at room temperature for 30 min compared with cheese that has been left for 90 min.

There are several limitations in this study. First, the small sample size in sensory evaluation (six panelists) and the evaluation based on one cheese may affect the score distribution and correlations. Second, the small number of tested cheese samples (six cheeses) may affect the sensory score distribution, e-nose values, and their correlations. Measurement of other types of cheeses (weaker aroma/stronger aroma) will expand the range of scores and values. Third, detection specificity of the metal oxide semiconductor sensor in e-nose and aroma collection method may affect the e-nose values. These limitations should be considered in future studies.

## 5. Conclusions

In conclusion, this study revealed correlations between mean e-nose values and the median of sensory evaluation scores of aroma intensity before intake, during mastication, and after swallowing, for the six types of cheese. An e-nose measures sample aroma non-destructively. In particular, the mean of e-nose values and the median of aroma intensity scores during mastication in sensory evaluation presented a linear relationship, and e-nose values may be useful in predicting cheese aroma intensity during mastication.

## 6. Patents

There is a pending patent in Japan related to the e-nose system used in this manuscript (Application No. 2020-192259).

## Figures and Tables

**Table 1:** Feature of 6 cheese samples.

Sample	A	B	C	D	E	F
Process/Natural	Process	Process	Process	Natural	Process	Process
Containing	-	-	Aged cheese	-	Blue cheese	Blue cheese

**Table 2:** Correlation coefficient among sensory scores (Median).

Sensory Evaluation	Rs/*p* Value	Before_Aroma	Mastication_Aroma	After_Aroma	Mastication_Taste	Lasting_Flavor
Before_Aroma	Rs	1.000	0.870 *	0.845 *	0.676	0.783
	*p* value		0.024	0.034	0.140	0.065
Mastication_Aroma	Rs	0.870 *	1.000	0.971 **	0.883 *	0.894 *
	*p* value	0.024		0.001	0.020	0.016
After_Aroma	Rs	0.845 *	0.971 **	1.000	0.886 *	0.883 *
	*p* value	0.034	0.001		0.019	0.020
Mastication_Taste	Rs	0.676	0.883 *	0.886 *	1.000	0.971 **
	*p* value	0.140	0.020	0.019		0.001
Lasting_Flavor	Rs	0.783	0.894 *	0.883 *	0.971 **	1.000
	*p* value	0.065	0.016	0.020	0.001	

Rs: Spearman’s rank correlation coefficient, * *p* < 0.05, ** *p* < 0.01.

## Data Availability

Data about personal sensory evaluation scores are not available due to contract restrictions.
